# Metal Ions in Activated Carbon Improve the Detection Efficiency of Aflatoxin-Producing Fungi

**DOI:** 10.3390/toxins11030140

**Published:** 2019-03-02

**Authors:** Tadahiro Suzuki, Masatoshi Toyoda

**Affiliations:** 1 Division of Food Biotechnology, Food Research Institute, NARO, 2-1-12 Kannon-dai, Tsukuba, Ibaraki 305-8642, Japan; 2 Food and Agricultural Materials Inspection Center (FAMIC), 2-1 Shin-toshin, Chuo, Saitama, Saitama 330-9731, Japan; masatoshi_toyoda498@famic.go.jp

**Keywords:** Aflatoxin, fluorescence, activated carbon, metal ion, detection

## Abstract

Aflatoxins (AF), produced by several *Aspergillus* species, are visible under ultraviolet light if present in high amounts. AF detection can be improved by adding activated carbon, which enhances the observation efficiency of weakly AF-producing fungi. However, commercial activated carbon products differ in their characteristics, making it necessary to investigate which characteristics affect method reproducibility. Herein, the addition of 10 activated carbon products resulted in different AF production rates in each case. The differences in the production of aflatoxin G_1_ (AFG_1_) were roughly correlated to the observation efficiency in the plate culture. Trace element analysis showed that the concentrations of several metal ions differed by factors of >100, and the carbons that most effectively increased AFG_1_ production contained higher amounts of metal ions. Adding 5 mg L^−1^ Fe or Mg ions increased AFG_1_ production even without activated carbon. Furthermore, co-addition of both ions increased AFG_1_ production stably with the addition of carbon. When varying the concentration of additives, only AFG_1_ production increased in a concentration-dependent manner, while the production of all the other AFs decreased or remained unchanged. These findings suggest that a key factor influencing AF production is the concentration of several metal ions in activated carbon and that increasing AFG_1_ production improves AF detectability.

## 1. Introduction

Aflatoxins (AFs), which are strong carcinogens, are secondary metabolites originating from *Aspergillus* fungi that can be present as contaminants in animal feed and various food products [1,2]. Each country has established reference values and strict controls for AFs in food processing and distribution to avoid contamination [2]. In recent years, climate change has led to the expansion of the distribution of AF-producing fungi from tropical to other regions, but the full extent of their distribution is unclear [3]. This is because the AF-producing fungi themselves are not targeted for regulation. In addition, a standard detection technique has not been established. Currently, the standard method for the detection of AF-producing fungi is polymerase chain reaction [4]. However, in the AF synthesis gene cluster, which contains the target region for the detection of AF-producing fungi, homologous or non-homologous recombination events occur throughout [5] and complicate detection. Therefore, other analyses such as ELISA, TLC, HPLC, and LC-MS are required to confirm the presence of AF-producing fungi. These tests rely on the detection of AFs rather than the fungi themselves. A sample solution must be prepared by the incubation of an isolated fungal strain, which makes it difficult to confirm all the AF-producing fungi, because fungi do not always produce AF.

Simple detection methods, such as plate cultivation, have also been developed. One method involves incubation on *Aspergillus flavus* and *Aspergillus parasiticus* agar, which contains aspergillic acid and Fe ions within colonies of aspergilli and is colored orange-brown [6]. This is a simple and relatively reliable method, although it cannot completely eliminate the possibility of false positives. Therefore, the application of this medium is thought to be mostly suitable as a first step for screening. Other methods are the ammonia addition and dichlorvos–ammonia methods—the latter being an improvement over the former [7,8]. These methods involve reacting an indicator with intermediates in the AF synthetic pathway, producing a deep peach color. Because color production is linked to the AF synthetic pathway, these methods are thought to have high reliability. However, the culture plate needs to be opened and closed to add ammonia, which is time-consuming compared with other methods. 

AFs themselves can also be detected, because they fluoresce under ultraviolet (UV) light [9]. However, this method is not commonly used, because it cannot detect low amounts of AFs. The cyclodextrin (CD) addition method has been developed to improve fluorescence detection [10,11]. The hydrophobic CD molecules, which are composed of sugar rings, form complexes with the AFs, which change their optical characteristics and drastically increase the AF fluorescence. Among the various CDs, α-CD, which is composed of six-membered sugar rings, has the highest fluorescence intensity in the presence of AF. This technique does not affect fungal growth and is often applied in screening studies [12,13]. However, it is still possible to miss fungi that produce low amounts of AF.

**Figure 1:**
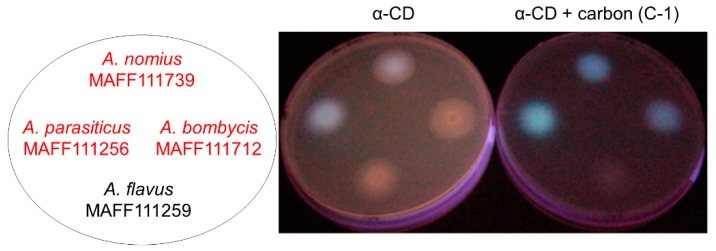
The addition of activated carbon improves the detection efficiency of aflatoxin-derived fluorescence. Fungal strains were cultured on potato dextrose agar (PDA) containing 3 g·L^−1^ α-CD (a type of cyclodextrin) and 0.3 g·L^−1^ activated carbon. Aflatoxin (AF)-derived fluorescence was observed on the bottom of the culture plate under UV irradiation. The fungal strains written in red produce AFs and those written in black do not.

A modified CD-addition method, which uses co-treatment with both CD and activated carbon, has been reported to improve the visibility of AF-derived fluorescence [14] (Figure 1). The improved visibility can be attributed to the fact that carbon addition reduces the reflection and scattering of UV light from the surface of the culture plate. In addition, carbon addition stimulates AF production, which increases AF fluorescence and improves its visibility. However, the mechanism by which carbon addition influences the fungal AF production remains unclear, as do the specific characteristics that induce the increase in production. The commercially available activated carbon products for this method have different characteristics (Table 1). This could adversely affect the reproducibility for this method.

In this study, to promote stable reproducibility of the carbon-addition method, we compared 10 activated carbon products from different suppliers and with differences in their raw materials, pH values, particle sizes, and activation methods. An activated carbon reagent that enhanced AF production and AF-derived fluorescence in an earlier study was labeled C-1 (Table 1). The trace metal ion compositions were also compared among the products in an attempt to identify specific factors that contributed to the reproducibility of the method.

## 2. Results

### 2.1. Comparative Analysis of the Addition of Each Carbon to the Culture Media

#### 2.1.1. Evaluation of Carbon Addition

**Figure 2:**
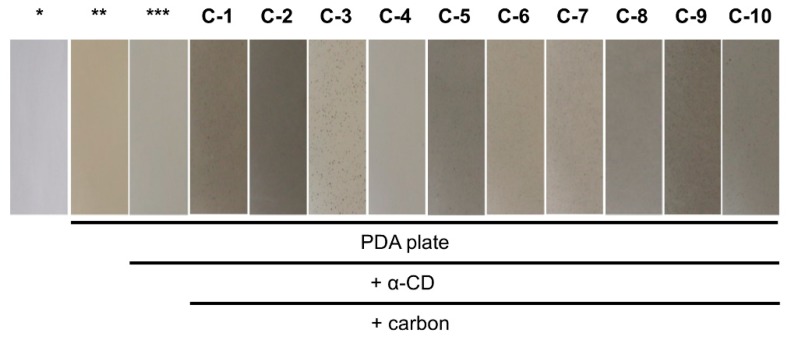
Colors of the culture plates with added activated carbon. * Background (no plate). ** PDA. *** PDA containing 3 g·L^−1^ α-CD.

In a preliminary evaluation of the 10 activated carbon products used to detect *Aspergillus bombycis* MAFF111712, we found large variation in the intensity of AF-derived fluorescence (Table 2). However, visual observations are subjective. Hence, to evaluate the general characteristics of each carbon additive, we prepared potato dextrose agar (PDA) culture plates containing 3 g·L^−1^ α-CD and 0.3 g·L^−1^ activated carbon and observed the color of the media (Figure 2). Several of the plates, including C-1, were a deep gray color, indicating decreased reflection and scattering of light. Plate C-4 contained a granular product that was not well dispersed, and the color was nearly the same as that of the PDA plate containing only α-CD. 

**Figure 3:**
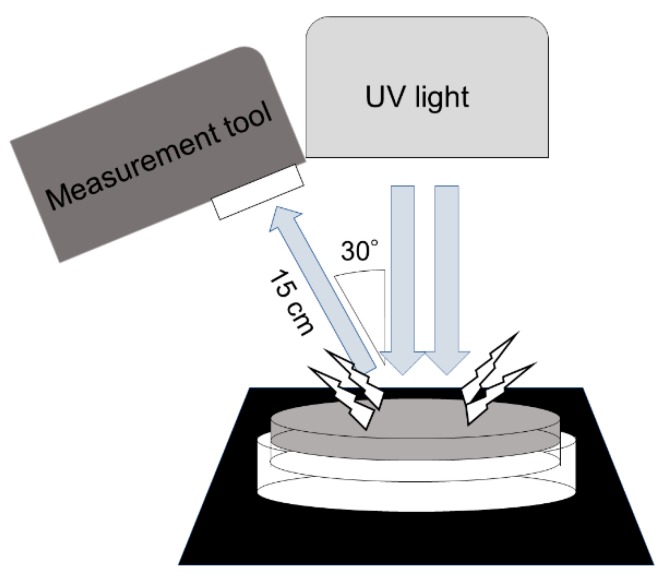
The procedure for measuring light scattering under UV irradiation.

To examine how the dispersion of carbon particles influenced the fluorescence visibility, we evaluated the intensity of the scattered light from the culture surface (Figure 3). To do this, we measured the photon flux density (PFD; μmol·m^−2^·s^−1^) in the visible region from 360 to 780 nm. All of the carbon-containing plates had lower PFDs than the plates containing only PDA or α-CD and PDA. Therefore, carbon addition decreased light scattering and improved the fluorescence visibility (Table 3). Among the plates, C-1, C-2, C-8, and C-9 showed very large decreases in light scattering. By contrast, the α-CD-containing PDA showed enhanced light scattering compared with the control PDA. These results were consistent with those from a previous study [14].

#### 2.1.2. Effects of Carbon Addition on Fungal Growth and AF Production

Next, we examined the effects of carbon addition on fungal growth and AF production using two fungal strains, *A. bombycis* MAFF111712 and *Aspergillus nomius* MAFF111739, which exhibited clear changes in fluorescence in the preliminary visual evaluation (Figure 1). For each activated carbon product, we prepared 500 μL of a culture solution containing potato dextrose (PD) and activated carbon. We did not include α-CD, because it does not influence the fungal growth but does drastically change the fluorescence. After a 5-day incubation, the fungal mass obtained with C-3 was significantly lower than that obtained with no carbon addition (*p* < 0.05, Table 4). With all the other activated carbon products, some differences were observed, but they were not significant. Only C-2, C-4, and C-5 gave the same level of fluorescence visibility as C-1, and among these activated carbon products, C-1 and C-5 were the most effective (Table 2). To investigate if the trends were the same in liquid and plate cultures, we conducted small-scale liquid culture tests using the tip culture method. With addition of C-1, C-2, C-4, and C-5, production of the green fluorescent AFG_1_ (“G” stands for green) increased significantly (*p* < 0.05, Table 5). By contrast, AFB_1_ production (“B” stands for blue) decreased with almost all of the activated carbon products. Some of the products caused significant differences in AFG_2_ and AFB_2_ production. However, these differences were small compared with those for AFG_1_ and AFB_1_, and only the AFG_1_ changes corresponded with visible differences in the fluorescence.

### 2.2. Analysis of Trace Element Composition

Because the different activated carbon products had different effects on AF production and the observable fluorescence on the culture plates, we investigated the characteristics of the carbon products to identify what caused these differences. However, an evaluation of the information available from the manufacturers of the products did not highlight anything that we thought could be related to the differences observed in AF production. Therefore, we presumed that another factor influenced the AF production. Although all the activated carbon products are composed of carbon, they are prepared from various raw materials and by different activation methods (Table 1). Therefore, they could have different trace element compositions, which could influence AF production. Thus, we used inductively coupled plasma optical emission spectroscopy (ICP-OES) and inductively coupled plasma mass spectrometry (ICP-MS) to investigate the trace element compositions of the activated carbon products (Table 6).

According to our analysis of 22 trace elements, including 21 metal ions and phosphorus, C-1, C-2, and C-5 had relatively high contents of a large number of these elements. By contrast, some activated carbon products, especially C-3 and C-7, which had unsatisfactory fluorescence signals in the preliminary observations, had low metal ion contents. Among the activated carbon products, C-1, C-2, and C-5, which detected AF-producing fungi most effectively, had the highest contents of Ca, Mg, Fe, Al, Mn, Ba, Sr, V, and Co. The contents of Ca, Mg, Fe, and Al were particularly high compared with the other products. C-1 and C-5, which were the most effective in the plate culture observation, had the highest contents of Al, Mn, Ba, Co, and Li. C-4, which was prepared from multiple raw materials and was granular rather than a powder, had low contents of all the elements compared with most other products, even though it produced relatively good observation in the plate culture experiment.

### 2.3. Effect of Trace Element Addition on AF Production

Because the AF-derived fluorescence improved with the addition of activated carbon products with high trace element contents, we suspected that these elements caused an increase in AF production. Hence, we investigated the effect of the direct addition of trace elements into the culture medium on the AF production. We separately added the three dominant elements that were common to C-1, C-2, and C-5 (Ca, Mg, and Fe) to the PD broth. Because most of the elements were assumed not to elute even during the autoclave sterilization process used to prepare each medium, the dilution rate was assumed to be 1000-fold and the concentrations were set to 5 mg·L^−1^. Our measurements of AF concentrations showed that AFG_1_ production from both *A. bombycis* MAFF111712 and *A. nomius* MAFF111739 increased upon the addition of Fe and Mg (Table 7). The addition of Ca only increased AFG_1_ production from *A. nomius* MAFF111739. Also, the addition of Fe and Mg decreased AFB_1_ production in *A. bombycis* MAFF111712 and *A. nomius* MAFF111739, respectively. Meanwhile, production of most other AFs did not change in either *A. bombycis* MAFF111712 or *A. nomius* MAFF111739 with addition of Ca, Mg, or Fe. The main result of this experiment was that Fe and Mg increased AFG_1_ production. Thus, these elements influence AF production when added to the basal culture medium (PD broth). However, the behavior of these elements in the activated carbon-containing medium was still unclear. Therefore, we selected C-3, which did not improve the fluorescence and did not increase the production of AFG_1_ or AFB_1_, for a growth study in the activated carbon-containing medium. In this study, we also tested the effects of Al, which was present at high levels in C-1, C-2, and C-5. Al was added to the C-3-containing medium at a concentration of 3 mg·L^−1^. On the basis of our earlier results, Ca was excluded from these tests. We found that AFG_1_ production by *A. bombycis* MAFF111712 increased upon the addition of Fe, and AFG_1_ production by *A. nomius* MAFF111739 increased upon the addition of Mg (Table 8). The addition of Al did not increase AF production.

Our results indicate that the trace elements Fe and Mg can exert valuable effects on AF production, but these effects are crucially dependent on the culture medium. The single addition of either of these metals may decrease the production of some AFs in activated carbon-containing media. 

**Figure 4:**
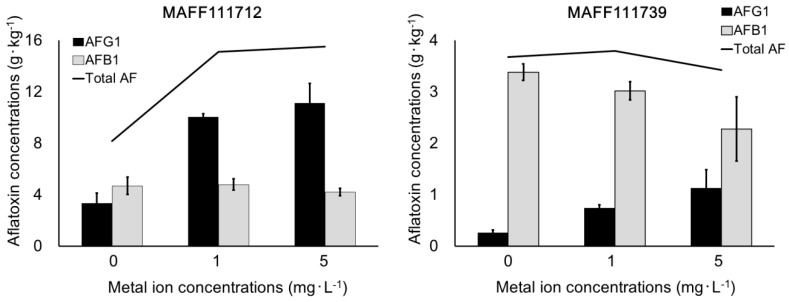
The influence of the addition of multiple trace elements on the C-3-containing culture. The final concentrations of Fe and Mg ions were set to 0, 1, or 5 mg·L^−1^, respectively. Bars = standard deviation (*n* = 4).

Next, we investigated the effect on AF production of the co-addition of Fe and Mg to the C-3-containing culture medium. We also evaluated the effect of changing the Fe and Mg concentration from 1 mg·L^−1^ to 5 mg·L^−1^. With the co-addition of these metals, AFG_1_ production increased in a concentration-dependent manner regardless of the fungal strain (Figure 4). Meanwhile, AFB_1_ production did not change or slightly decreased.

## 3. Discussion

Various activated carbon products with the same name but different characteristics are available commercially. In this study, we compared 10 of these products (Table 1) with respect to their effect on AF production. C-1, produced by the steam activation of peat, is a pH-neutral activated carbon that has been previously studied in this context [14]. Although some of the other as-obtained products were not pH-neutral, their acidities were equalized by preparing the culture medium. Therefore, it is unlikely that the pH of each carbon reagent affected the AF production or fluorescence visibility. Hence, if the factor(s) that caused the differences in the AF production and fluorescence visibility were included in the manufacturers’ product information, the raw material, activation method, and particle size are the potential candidates. Looking for the similarity between C-1, C-2, C-4, and C-5, which all displayed increased visual fluorescence signals, it can be found that all four reagents were produced by steam activation. This raises the possibility that reagents prepared by steam activation are particularly effective. In contrast, there are no patterns in the raw material or particle size. However, the addition of C-7, which was also produced by steam activation, did not contribute to the enhancement of the fluorescence signal. We could not obtain the activation method of C-9. Meanwhile, the addition of C-3, C-8, and C-10, which were prepared by acid activation, resulted in small changes or decreases in the AF production and did not enhance the fluorescence visibility. From these data, it can be inferred that reagents produced by steam activation show a promising tendency to enhance the detectability of AF-producing fungi.

The content of trace elements in the raw materials is likely to be the factor most influenced by the difference in activation methods. Therefore, we investigated the trace element compositions of the 10 products. Trace elements were abundant in C-1, C-2, and C-5, and much scarcer in C-3, C-8, and C-10. This suggests that, despite the exceptions and incomplete information found in this study, the activation method will be, to some extent, a helpful consideration in selecting activated carbon reagents. Meanwhile, for C-4, which enhanced the fluorescence visibility of *A. bombycis* MAFF111712 despite its low content of trace elements, the data indicated a distinct set of characteristics compared with all the other products. C-4 was the only product in which coal was listed as one of the raw materials, and the product was prepared by a different process than all the others [15]. Therefore, a different approach is required to predict the effect of the C-4 addition.

When we investigated the relationship between the AF volumes in the liquid culture study and the fluorescence intensities in the plate culture study, we found that only AFG_1_ production corresponded to the fluorescence intensities observed in the preliminary plate test (Table 2 and Table 5). The other AFs were not correlated with the fluorescence intensities, except for AFB_1_, which actually showed the inverse trend compared with the plate fluorescence. An increase in the production of AFG_1_, an AF with green fluorescence, appeared to be a common factor resulting in high fluorescence. Green wavelengths are longer than blue and are more visible to the human eye, which is more sensitive to this color than any other [16,17]. Consequently, we propose that the addition of activated carbon improves visibility by increasing the production of AFs with green fluorescence, even if the total AF volume does not change.

The trace element compositions of the activated carbon products were determined by ICP-OES and ICP-MS (Table 6), and several trace ions showed differences of more than 100-fold among the carbon products. Activated carbon is mainly used in adsorption applications and its trace element composition is not usually investigated. The idea that trace elements contained in activated carbon products may affect the production of fungal secondary metabolites is novel and requires further investigation. In this study, we only investigated 22 trace elements, and others should be considered in future studies. Our results showed high contents of some trace elements (i.e., P, K, Ca, Mg, Fe, and Al) in the activated carbon products. We excluded P and K from our subsequent investigations because of their low contents in C-1 and C-5. Because Ca ions are important in the cellular ion transport system, we investigated Ca first [18] (Table 7). However, the changes induced by Ca addition were complicated, and these investigations did not produce any useful information. Accordingly, we next focused on Mg, Al, and Fe. Because the elution efficiency of each activated carbon product was unknown, the trace element concentrations for the addition experiments were selected randomly. However, fortuitously, the concentrations were similar to those used in earlier studies [19,20]. Those studies reported that the addition of Co, Zn, Mn, and Cu increased AF production, and among these elements, Zn was the most promising. However, in the present study, we could not confirm the relationship between Zn and AF production because of the low concentrations of this metal. 

To confirm the effect of Mg, Al, and Fe, we conducted a metal ion addition test with C-3, which contained lower levels of metal ions than the other activated carbon products. The results demonstrated that the addition of Fe and Mg ions increased AF production (Table 8). This effect would appear to be consistent with the well-known success of Czapek medium, a standard fungal culture medium that contains Fe and Mg as trace elements [21]. However, excessively high concentrations of metal ions are known to decrease AF production [19,22]. The Fe and Mg concentrations in Czapek medium are 7 to ten times those described in previous studies of AF production [19,20]. Therefore, this solution would not be suitable for inducing AF production. Thus, the optimum concentration to increase AF production still has to be determined in this study. In a short-term incubation experiment using the same conditions as that of PDA, Czapek medium containing activated carbon and CD did not induce adequate AF-derived fluorescence because of insufficient colony formation, so Czapek medium was not selected as the base medium in this study. The final concentrations of Fe and Mg were set to 1 and 5 mg·L^−1^, respectively, to determine the optimum concentration, and we found that 5 mg·L^−1^ maximized AFG_1_ production. By contrast, the production of AFB_1_, AFB_2_, and AFG_2_ with 5 mg·L^−1^ Fe and Mg was lower than with 1 mg·L^−1^ Fe and Mg. These results show that the addition of trace elements at 5 mg·L^−1^ has a negative effect on some AFs. Additionally, our results suggest that the co-addition of Fe and Mg provides a more reproducible effect on the production of AFG_1_ than the addition of a single element. The increase in AF production with the activated carbon addition might have been caused by the presence of multiple co-existing metal ions. Taking into consideration all of the above results, the addition of up to 5 mg·L^−1^ Fe and Mg is useful for increasing the intensity of AF-derived fluorescence even in an unsatisfactory culture medium with weak fluorescence. Our results indicate that both AF concentrations and AF-derived fluorescence increase with the addition of trace elements. However, the trace elements themselves do not decrease light scattering on the surface of the culture plate. Addition of any activated carbon product will decrease light scattering (Table 3) and will not inhibit fungal growth in the activated carbon-containing culture medium unless there is an extreme lack of trace elements (Table 4). In summary, the co-treatment of the culture medium with an activated carbon product and trace metal ions will produce better results than treatment with only trace metal ions.

It was unclear why only the production of G-type AFs was enhanced by the addition of metal ions. In an earlier study, we likewise found that carbon addition increased the production of G-type AFs [14]. However, this phenomenon was only observed in strains that produce both B and G-type AFs, such as *A. parasiticus*, which was attributed to the *nadA* gene, located downstream of the AF synthesis gene cluster. Yabe et al. reported that the *nadA* gene encodes NADH or NADPH oxidase, which converts the intermediate of AFB_1_ to AFG_1_ [23]. Some reports suggest that the activation of NADPH oxidase is regulated by Mg ions through various processes [24,25,26]. From these findings, we can hypothesize that an increase in some metal ions in the culture medium will activate the enzyme and increase AFG_1_ production. In support of this hypothesis, the addition of Fe and Mg did not change the total quantity of AFs synthesized by the *A. nomius* MAFF111739 strain but did increase the proportion of AFG_1_. Meanwhile, these ions induced few changes in AFB_1_ production by *A. bombysis* MAFF111712, although AFG_1_ production increased. These changes in the composition of AFs can be partly attributed to the differences among the activated carbon products (Table 5). Additionally, in an earlier study using *A. flavus* strains that produce only B-type AFs, activated carbon addition increased the production of AFB_1_ and AFB_2_ [14]. Activated carbon products contain various trace elements and their addition affects fungal growth, but there may be other characteristics that also contribute to the changes observed in AF production. Further investigation of activated carbons is required to understand the mechanism that results in improved AF production.

## 4. Conclusions

The addition of any activated carbon can be assumed to decrease light scattering on the culture plate. However, this study further demonstrated that carbon products containing an abundance of certain metal ions, such as Fe and Mg, increase AF production and improve the visibility of their fluorescence signal. Our results provide some insight into how the addition of activated carbon into a culture improves AF-derived fluorescence, and this knowledge could be used to improve the reproducibility of the carbon addition method. The combination of any activated carbon and less than 5 mg·L^−1^ metal ions can be expected to improve the detection conditions of AF-producing fungi. However, it is difficult to obtain relevant information about commercial activated carbon products to prepare the appropriate levels of metal ion concentrations that can stably and reproducibly increase both AF production and fluorescence visibility. The development of a master mixture of trace elements or a premix reagent will be required for the popularization of this technique.

## 5. Materials and Methods

### 5.1. Fungal Species

The fungal strains *A. flavus* MAFF111259, *A. parasiticus* MAFF111256, *A. bombycis* MAFF111712, and *A. nomius* MAFF111739 were obtained from the Genebank Project, National Agriculture and Food Research Organization (Ibaraki, Japan). Each sample was cultured on a PDA (Merck, Darmstadt, Germany) slant, and incubated at 28 °C for several days. Spores were collected using 0.05% Tween 80 (ICN Biomedicals, Santa Ana, CA, USA) and stored at 4 °C

### 5.2. Culture Conditions and Apparatus

For observing AF-derived fluorescence, we prepared PDA supplemented with 3 g·L^−1^ α-CD (FUJIFILM Wako Pure Chemical, Osaka, Japan) and 0.3 g·L^−1^ activated carbon (Table 1). We also prepared PD broth (Merck) with activated carbon and without α-CD as the liquid culture medium. Aliquots (495 μL) of the liquid medium were dispensed into 1-mL pipette tips that were sealed with laboratory film (PM-996, Bemis, Neenah, WI, USA) and used for incubation [27]. The total volume was adjusted to 500 μL by adding the spore solution. To prepare trace element solutions, (CH_3_CHOCOO)_2_Ca·5H_2_O, MgSO_4_·7H_2_O, FeCl_3_·6H_2_O, and [CH_3_CH(OH)COO]_3_Al (FUJIFILM Wako Pure Chemical) were dissolved in distilled water. The trace element solutions were added to the liquid culture media. The final concentrations of Ca, Mg, and Fe were set to 5 mg·L^−1^ and that of Al was set to 3 mg·L^−1^. The fungal spore solution (5 μL) was dispensed onto a culture plate or into the liquid culture medium. After liquid cultivation, the laboratory film was removed from each pipette tip, and the tips were centrifuged at 3000× *g* for 15 min. The AF concentrations were measured in the obtained culture solutions. Mycelia with spores in the incubation tip were dried in a dehydrator (SLI-450N, TOKYO RIKAKIKAI, Tokyo, Japan) at 65 °C overnight, and the fungal mass was calculated using the tip masses before and after incubation.

### 5.3. Measurement of Light Scattering

The spectrum of reflected and scattered light was measured by an illuminance spectrophotometer (CL-500A; Konica Minolta, Tokyo, Japan) as the irradiance (W·m^−2^). The total spectrum from 360 to 780 nm was measured, and the PFD for each spectral condition was calculated using the following formula: PFD (μmol·m^−2^·s^−1^) = [irradiance (W·m^−2^) × spectrum (m) × 10^−9^]/[Planck’s constant (6.626 × 10^−34^; J·s) × speed of light (2.998 × 10^8^; m·s^−1^) × Avogadro’s constant (6.022 × 10^23^; mol^−1^)] × 10^6^. The PFD results were used to compare PDA plates with and without α-CD and activated carbon. The bottom of each culture plate was irradiated with UV light at 365 nm (UVGL-58, UVP, Upland, CA, USA) in a dark room. The plates for each sample condition were prepared at least in triplicate.

### 5.4. High-Performance Liquid Chromatography

The liquid culture medium and an equivalent volume of chloroform were dispensed into new microtubes and vortexed for 10 s. The chloroform layer was collected and transferred to a new microtube and then reduced to dryness in a draft chamber at 40 °C. The subsequent procedures followed the official method [28]. In this study, the limits of quantitation and detection were 2 μg·kg^−1^ and 1 μg·kg^−1^, respectively. An aliquot of trifluoroacetic acid (Wako Pure Chemical Industries) equal to 0.1 times the volume of the chloroform was added to the microtube to derivatize AFB_1_ and AFG_1_ with vortexing for 5 s. After incubation for more than 10 min, a mixture of acetonitrile in distilled water (10:90, *v/v*) was added to the microtube. The volume of acetonitrile/water was 0.9 times the volume of chloroform. An aliquot (20 µL) of the sample solution was injected into a high-performance liquid chromatography system (SCL-10A, Shimadzu, Kyoto, Japan) equipped with a fluorescence detector (λ_Ex_ = 365 nm and λ_Em_ = 455 nm; RF-535, Shimadzu). The mobile phase was a mixture of distilled water/methanol/acetonitrile (60:30:10, *v/v/v*) with a flow rate of 1 mL·min^−1^. The column was an Inertsil ODS-3 (150 mm × 4.6 mm, particle size 5 μm; GL Sciences, Tokyo, Japan).

### 5.5. ICP-OES and ICP-MS Analyses

About 0.05 g of each sample was weighed into a polytetrafluoroethylene beaker. Then, 5 mL or more of 61% HNO_3_ was added into the beaker. A polytetrafluoroethylene watch glass was placed on the beaker, and the beaker was heated to 80–150 °C on a hot plate for 3 h. After heating, 1.0 mL of 70% HClO_4_ was added, and the beaker was heated to 230 °C for 8–30 h. After cooling, 1.0 mL of 48% HF was added, and the beaker was heated to 100 °C for 4 h. Then, 0.5 mL of H_2_O_2_ was added, and the beaker was heated at 80–100 °C for 2 h. The watch glass was removed and the beaker was heated continuously until all the solvent evaporated. The residue was dissolved in 1% nitric acid, placed in a 50 mL volumetric flask with 0.25 mL of a 1 mg·L^−1^ indium solution, and then diluted to the mark with 1% nitric acid. The concentrations of 13 elements (Na, Mg, Al, P, K, Ca, V, Cr, Mn, Fe, Zn, Sr, and Ba) were determined by ICP-OES (Agilent 5100, Agilent Technologies, Santa Clara, CA, USA), and 9 elements (Li, Be, Co, Ni, Cu, Rb, Ag, Cd, and Pb) were determined by ICP-MS (Thermo Fisher Scientific ELEMENT 2, Thermo Fisher Scientific, Waltham, MA, USA).

### 5.6. Statistical Analyses

We calculated average values and standard deviations for scattered light intensities, fungal masses, and AF levels. All the comparisons were conducted using *F*-tests, and equally distributed conditions (*p* < 0.05) were analyzed using unpaired *t*-tests.

## Figures and Tables

**Table 1:** Available information about the commercial activated carbon products.

Set No.	Description	Cat. No. ^1^	Particle size	Material, Activation Method	pH	Supplier
**C-1**	Powder, Neutral	031-18103	<150 μm	Peat, Steam activation	6.0~7.2	FUJIFILM Wako Pure Chemical
**C-2**	Powder, Basic	032-18091	<150 μm	Peat, Steam activation	10	FUJIFILM Wako Pure Chemical
**C-3**	Powder	031-02135	<300 μm	Sawn wood, Acid washed	6.0	FUJIFILM Wako Pure Chemical
**C-4**	Granule	01084-12	3.35~4.75 mm	Coal, Coconut shell, Steam activation	6.0~8.0	Kanto Chemical
**C-5**	Powder	08304-08	<75 μm	Sawn wood, Steam activation	9.0~11.0	Kanto Chemical
**C-6**	Powder	08305-08	<150 μm	Coconut shell, Steam activation	6.0~8.0	Kanto Chemical
**C-7**	Powder	01085-02	20 μm	Sawn wood, Steam activation	6.0~8.0	Kanto Chemical
**C-8**	Powder	07909-65	<40 μm	Sawn wood, Acid washed	5.0~8.0	Nacalai tesque
**C-9**	Powder, Darco G-60	537-02241	45~150 μm	Natural product, <10 % silica, ^2^	6.0~8.0	FUJIFILM Wako Pure Chemical
**C-10**	Powder, Norit(R) A pract.	30890.01	45~75 μm	Peat, Acid washed	6.0~8.0	FUJIFILM Wako Pure Chemical

^1^ Cat. No.: catalogue number. ^2^ No information was available about the activation method for C-9.

**Table 2:** Preliminary evaluation of the aflatoxin-derived fluorescence observed with different activated carbon products.

PDA with 3 g·L^−1^ α-CD										
—	C-1	C-2	C-3	C-4	C-5	C-6	C-7	C-8	C-9	C-10
±	+++	++	±	++	+++	+	±	+	+	+

Visual characteristics of fluorescence: ±, not detected; +, measurable; ++, clear; and +++, bright. Conditions: 28 °C and 2-day incubation.

**Table 3:** Changes in the ratios of the measured photon flux densities (%).

*	α-CD										
	**	C-1	C-2	C-3	C-4	C-5	C-6	C-7	C-8	C-9	C-10
100 ± 2.3	145.9 ± 1.9	13.3 ± 1.1	12.8 ± 2.9	51.4 ± 2.4	83.5 ± 2.4	45.0 ± 1.6	29.8 ± 1.3	54.2 ± 10.7	22.7 ± 1.8	−15.4 ± 2.7	31.5 ± 1.8

* PDA. ** 3 g·L^−1^ α-CD-containing PDA. The results are the average ± standard deviation (*n* = 4).

**Table 4:** Mycelia masses (mg) of *Aspergillus* strains obtained in small-scale liquid cultures.

	— *	C-1	C-2	C-3	C-4	C-5	C-6	C-7	C-8	C-9	C-10
**MAFF** **111712**	1.75 ± 0.13	1.72 ± 0.25	1.58 ± 0.12	1.35 ± 0.36	1.50 ± 0.17	1.92 ± 0.19	1.62 ± 0.12	1.71 ± 0.33	1.53 ± 0.24	1.69 ± 0.23	1.77 ± 0.37
**MAFF** **111739**	2.04 ± 0.38	2.33 ± 0.19	2.28 ± 0.21	1.42 ± 0.21	1.73 ± 0.21	2.45 ± 0.36	1.92 ± 0.23	1.97 ± 0.16	1.57 ± 0.34	2.25 ± 0.14	2.11 ± 0.27

* potato dextrose (PD), C-1–C-10; PD with 0.3 g·L^−1^ activated carbon. The results are the average ± standard deviation (*n* ≥ 3).

**Table 5:** Aflatoxin production in small-scale liquid cultures.

***Aspergillus bombycis* MAFF111712**											
	— *	**C-1**	**C-2**	**C-3**	**C-4**	**C-5**	**C-6**	**C-7**	**C-8**	**C-9**	**C-10**
**AFG1**	5.44 ± 0.69	8.28 ± 1.04	6.25 ± 1.63	4.11 ± 0.21	7.67 ± 1.22	5.97 ± 0.44	6.70 ± 1.60	5.91 ± 1.67	5.26 ± 0.81	6.72 ± 1.58	5.51 ± 0.62
**AFB1**	4.05 ± 0.53	3.55 ± 0.97	2.90 ± 0.66	3.00 ± 0.64	3.62 ± 0.35	2.75 ± 0.11	3.49 ± 0.50	3.42 ± 0.47	2.33 ± 0.49	3.14 ± 0.79	2.96 ± 0.18
**AFG2**	0.06 ± 0.01	0.06 ± 0.01	0.05 ± 0.01	0.06 ± 0.01	0.10 ± 0.01	0.07 ± 0.02	0.11 ± 0.02	0.10 ± 0.04	0.11 ± 0.03	0.14 ± 0.06	0.12 ± 0.03
**AFB2**	0.10 ± 0.01	0.05 ± 0.01	0.05 ± 0.01	0.09 ± 0.03	0.10 ± 0.00	0.07 ± 0.02	0.13 ± 0.03	0.13 ± 0.03	0.10 ± 0.02	0.13 ± 0.05	0.13 ± 0.03
***Aspergillus nomius* MAFF111739**											
	— *	**C-1**	**C-2**	**C-3**	**C-4**	**C-5**	**C-6**	**C-7**	**C-8**	**C-9**	**C-10**
**AFG1**	0.18 ± 0.03	0.31 ± 0.12	0.30 ± 0.09	0.18 ± 0.08	0.49 ± 0.03	0.38 ± 0.18	0.26 ± 0.05	0.18 ± 0.07	0.22 ± 0.09	0.15 ± 0.04	0.13 ± 0.03
**AFB1**	1.60 ± 0.24	0.59 ± 0.19	0.43 ± 0.11	1.55 ± 0.48	1.91 ± 0.10	1.02 ± 0.38	1.23 ± 0.06	1.10 ± 0.44	1.01 ± 0.31	0.57 ± 0.08	0.69 ± 0.16
**AFG2**	0.00 ± 0.00	0.00 ± 0.00	0.00 ± 0.00	0.00 ± 0.00	0.00 ± 0.00	0.00 ± 0.00	0.00 ± 0.00	0.00 ± 0.00	0.00 ± 0.00	0.00 ± 0.00	0.00 ± 0.00
**AFB2**	0.01 ± 0.00	0.00 ± 0.00	0.00 ± 0.00	0.02 ± 0.01	0.01 ± 0.00	0.01 ± 0.00	0.01 ± 0.00	0.01 ± 0.01	0.02 ± 0.01	0.01 ± 0.00	0.01 ± 0.00

* PD, C-1–C-10; PD with 0.3 g·L^−1^ carbon. The results are the average ± standard deviation (mg·kg^−1^, *n* ≥ 3).

**Table 6:** Metal ions in the commercial activated carbon products (mg·kg^−1^).

Set No.	P	K	Ca	Mg	Fe	Al	Na	Mn	Zn	Ba	Sr
**C-1**	10206	1451	9251	5484	6452	3801	932	140	5.95	107	96.3
**C-2**	1081	756	9397	8970	6662	2678	693	83.3	1.44	80.5	97.4
**C-3**	N.D.	3.9	117	62.8	51	33.1	12.4	0.9	242	0.97	1.05
**C-4**	97.6	541	77.7	41.3	325	264	73.8	6.74	9.98	3.09	1.31
**C-5**	742	12483	8205	1823	2100	3953	375	404	9.23	89.5	49.6
**C-6**	140	1308	200	179	1025	914	293	13.3	5.69	5.54	3.61
**C-7**	22.2	132	108	47	112	371	46	4.09	6.66	4.38	1.37
**C-8**	14703	3245	982	393	602	491	417	38.6	8.96	6.95	6.26
**C-9**	347	260	900	1042	1176	2381	423	18.2	1.9	29.5	15.4
**C-10**	166	344	1223	630	453	1048	261	14.6	2.6	21.3	8.6
Detection limit	5.6	1.7	16	0.77	3.5	14	3.4	0.27	0.56	0.41	0.02
**Set No.**	**Cr**	**Rb**	**Cu**	**V**	**Ni**	**Pb**	**Co**	**Li**	**Ag**	**Be**	**Cd**
**C-1**	60.7	3.35	11.6	15.6	6.57	2.2	2.81	2.56	0.03	0.91	0.02
**C-2**	7.21	2.4	7.2	7.43	3.03	0.12	0.97	0.6	0.01	0.08	0.01
**C-3**	4.89	0.02	3.32	0.74	2.11	11.7	0.3	N.D. *	0.03	0.28	0.02
**C-4**	5.79	3.23	30.5	2.09	4.35	0.26	0.37	0.17	0.01	0.01	0.01
**C-5**	4.23	62.4	16.8	4.86	3.53	1.09	1.1	4.5	0.02	0.14	0.03
**C-6**	62.5	3.6	19.2	1.25	15.1	0.22	0.67	0.8	0.1	0.05	0.02
**C-7**	13.9	0.82	12.5	N.D. *	3.45	0.21	0.13	0.17	0.24	0.04	0.02
**C-8**	12.9	13.4	17	0.89	4.99	1.09	0.3	0.27	0.05	0.02	0.03
**C-9**	7.34	0.85	8.86	2.65	3.73	0.21	0.84	0.29	0.02	0.1	0.01
**C-10**	50.4	1.1	8.85	1.54	7.36	0.31	0.5	0.41	0.02	0.04	0.02
Detection limit	0.5	<0.01	0.19	0.47	0.08	0.04	<0.01	0.01	<0.01	<0.01	<0.01

* Less than the detection limit or not detected.

**Table 7:** Test using the addition of trace metal ions present at high levels in activated carbon.

*A. bombycis* MAFF111712					*A. nomius* MAFF111739				
	PD ^1^					PD			
	— ^1^	Ca ^2^	Fe ^2^	Mg ^2^		—	Ca	Fe	Mg
**AFG1**	5.44 ± 0.69	4.72 ± 0.78	7.06 ± 1.32	9.89 ± 0.02	**AFG1**	0.18 ± 0.03	0.25 ± 0.07	0.28 ± 0.05	0.35 ± 0.09
**AFB1**	4.05 ± 0.53	3.00 ± 0.46	2.85 ± 0.47	4.93 ± 0.14	**AFB1**	1.60 ± 0.24	1.55 ± 0.32	1.67 ± 0.30	1.03 ± 0.24
**AFG2**	0.06 ± 0.01	0.05 ± 0.01	0.07 ± 0.01	0.08 ± 0.01	**AFG2**	0.00 ± 0.00	0.00 ± 0.00	0.00 ± 0.00	0.00 ± 0.00
**AFB2**	0.10 ± 0.01	0.07 ± 0.01	0.06 ± 0.01	0.08 ± 0.01	**AFB2**	0.01 ± 0.00	0.01 ± 0.00	0.01 ± 0.00	0.01 ± 0.00

^1^ Basal culture media. ^2^ The metal ion concentration was set to 5 mg·L^−1^. The results are presented in mg·kg^−1^ and are the average ± standard deviation (*n* ≥ 3).

**Table 8:** Trace metal ion addition in the presence of activated carbon.

*A. bombycis* MAFF111712					*A. nomius* MAFF111739				
	PD with 0.3 g·L^−1^ C-3^1^					PD with 0.3 g·L^−1^ C-3			
	— ^1^	Ca ^2^	Fe ^2^	Mg ^2^		—	Ca	Fe	Mg
**AFG1**	5.44 ± 0.69	4.72 ± 0.78	7.06 ± 1.32	9.89 ± 0.02	**AFG1**	0.18 ± 0.03	0.25 ± 0.07	0.28 ± 0.05	0.35 ± 0.09
**AFB1**	4.05 ± 0.53	3.00 ± 0.46	2.85 ± 0.47	4.93 ± 0.14	**AFB1**	1.60 ± 0.24	1.55 ± 0.32	1.67 ± 0.30	1.03 ± 0.24
**AFG2**	0.06 ± 0.01	0.05 ± 0.01	0.07 ± 0.01	0.08 ± 0.01	**AFG2**	0.00 ± 0.00	0.00 ± 0.00	0.00 ± 0.00	0.00 ± 0.00
**AFB2**	0.10 ± 0.01	0.07 ± 0.01	0.06 ± 0.01	0.08 ± 0.01	**AFB2**	0.01 ± 0.00	0.01 ± 0.00	0.01 ± 0.00	0.01 ± 0.00

^1^ Basal culture media, ^2^ The metal ion concentration was set to 5 mg·L^−1^. ^3^ The metal ion concentration was set to 3 mg·L^−1^. The results are presented in mg·kg^−1^ and are the average ± standard deviation (*n* ≥ 3).
